# Using genomics to characterize evolutionary potential for conservation of wild populations

**DOI:** 10.1111/eva.12149

**Published:** 2014-03-14

**Authors:** Katherine A Harrisson, Alexandra Pavlova, Marina Telonis-Scott, Paul Sunnucks

**Affiliations:** 1 School of Biological Sciences, Monash UniversityMelbourne, Vic., Australia

**Keywords:** climate change, genetic variation, genome-wide diversity, local adaptation, natural selection, polygenic adaptation, population persistence, wildlife management

## Abstract

Genomics promises exciting advances towards the important conservation goal of maximizing evolutionary potential, notwithstanding associated challenges. Here, we explore some of the complexity of adaptation genetics and discuss the strengths and limitations of genomics as a tool for characterizing evolutionary potential in the context of conservation management. Many traits are polygenic and can be strongly influenced by minor differences in regulatory networks and by epigenetic variation not visible in DNA sequence. Much of this critical complexity is difficult to detect using methods commonly used to identify adaptive variation, and this needs appropriate consideration when planning genomic screens, and when basing management decisions on genomic data. When the genomic basis of adaptation and future threats are well understood, it may be appropriate to focus management on particular adaptive traits. For more typical conservations scenarios, we argue that screening genome-wide variation should be a sensible approach that may provide a generalized measure of evolutionary potential that accounts for the contributions of small-effect loci and cryptic variation and is robust to uncertainty about future change and required adaptive response(s). The best conservation outcomes should be achieved when genomic estimates of evolutionary potential are used within an adaptive management framework.

## Introduction

The rapid pace of human-driven global environmental change is a well-recognized threat to global biodiversity (Rands et al. 2010). Factors such as climate change, habitat fragmentation and environmental degradation are influencing the distribution and abundance of species through many direct and indirect effects that are often difficult to predict (Bellard et al. 2012). Thus, a central question in conservation is how best to manage for species persistence under rapidly changing and often unpredictable environmental conditions (Hoffmann and Sgrò 2011).

When faced with environmental change, species may persist by moving (or being moved) to track suitable environments. However, whilst tracking environments spatiotemporally can be an important response to environmental change, concomitant factors (e.g. habitat loss and fragmentation) can limit the effectiveness of the response (Mantyka-Pringle et al. 2012). Poorly dispersing organisms and/or habitat specialists may be unable to shift their ranges in response to changing conditions (Schloss et al. 2012). Whilst movement can be promoted through reconnection of habitat or via active translocations (Weeks et al. 2011), recent analyses suggested that the extent to which such interventions will be necessary for vertebrates may impose infeasible burdens on ecological management agencies (Vander Wal et al. 2013).

Alternatively, populations can respond to environmental change either within the lifetimes of individuals through plasticity (i.e. phenotypic changes that do not depend on immediate heritable genetic change) or over multiple generations through evolutionary adaptation. There is increasing recognition of, and experimental evidence for, the importance of plastic responses to climate change (Chevin et al. 2010). Phenotypic plasticity can be a rapid and profound mechanism for biota to suit their environments better, without incurring the demographic costs of natural selection (Reed et al. 2011). This may be particularly true for longer-lived species whose rate of evolutionary adaptation will be slow, owing to long generation times (Chevin et al. 2010; Vander Wal et al. 2013). However, plasticity is often associated with fitness costs (Chevin et al. 2010). For this reason, whilst plasticity may offer an important stopgap measure for populations facing environmental change, evolutionary adaptations over multiple generations will still be essential for ensuring persistence into the future (Bradshaw and Holzapfel 2006), especially when environmental change occurs over a large area and long period of time (e.g. climate change; Lynch and Lande 1993). Importantly, plasticity and evolutionary adaptation are not mutually exclusive; plasticity has a genetic basis and can evolve (Bijlsma and Loeschcke 2012). A growing body of theoretical and empirical evidence supports the idea that populations can rapidly evolve under environmental change (e.g. Bell and Gonzalez 2009; Rodriguez-Trelles et al. 2013; but see Gienapp et al. 2013). Thus, promoting evolutionarily resilient species and ecological communities in which evolutionary potential (i.e. capacity to evolve in response to changing environments) is maximized is increasingly recognized as a conservation necessity (Sgrò et al. 2011).

Small sets of purportedly neutral, anonymous markers traditionally used in conservation management (e.g. mitochondrial DNA and/or tens of microsatellite markers) have not proven good predictors of genome-wide diversity and/or evolutionary potential (Reed and Frankham 2001). With the advent of next-generation sequencing technologies (high-throughput, massively parallel sequencing; Appendix S1), it is now possible to apply genomic methods to nonmodel organisms and screen large numbers of individuals for large numbers (thousands to tens of thousands) of genome-wide markers at relatively low cost (Cosart et al. 2011; Bi et al. 2012; Lemmon et al. 2012). Thus, genomics has quickly become an important and rapid conservation tool to explore evolutionary processes relevant to population persistence: inbreeding depression, outbreeding depression, hybridization, introgression and adaptation (e.g. Allendorf et al. 2010; Angeloni et al. 2012).

Here, we explore the strengths and limitations of genomics as a tool for characterizing evolutionary potential in nonmodel organisms. Our goal is to summarize some of the complexity of adaptation genetics and to outline the implications of this complexity for deriving estimates of evolutionary potential from genomic sequence data. More specifically, we (i) outline challenges associated with estimating evolutionary potential in wild populations, (ii) summarize knowledge of the genetic and epigenetic components of evolutionary potential, (iii) review current methods and techniques for characterizing adaptively important variation, (iv) develop recommendations for estimating evolutionary potential in a management context, and (v) outline future research directions. Whilst our primary focus is restricted to genomics as a tool for estimating evolutionary potential in wild populations, we would also direct readers to exciting advances in the parallel field of phenomics which, through use of high-dimensional phenotype data, offers an alternative, nongenetic approach to estimating evolutionary potential (see e.g. Houle et al. 2010).

### Limitations of traditional quantitative genetic approaches prompt supplementary and/or alternative methods for characterizing evolutionary potential in wild populations

For single traits, short-term evolutionary potential is contingent on the additive genetic variance associated with a trait in a population. Estimating additive genetic variance requires partitioning observed phenotypic variation into its genetic (additive and nonadditive) and environmental components. To allow comparison across traits and populations, measures of additive genetic variance must be standardized (Houle 1992; Hansen et al. 2011). In quantitative genetics, narrow-sense heritability measures the proportion of phenotypic variance that is additive and hence is a variance-standardized measure of additive genetic variation that indicates the extent to which a trait is genetically determined (Houle 1992; Hansen et al. 2011). Despite its common use as a trait-based proxy for evolutionary potential (Visscher et al. 2008), in many situations narrow-sense heritability may have little correlation with either actual capacity for rapid evolution in natural populations or with genetic variability, most likely because of inherent positive correlations between additive genetic variance and other components of phenotypic variance (Houle 1992; Hansen et al. 2011). As a consequence, traits with low heritability can still have high evolutionary potential. Houle (1992) proposed ‘evolvability’ (termed the additive genetic coefficient of variation) as a more appropriate measure of evolutionary potential, where additive genetic variance is standardized using the population trait mean and hence made independent of other variance components (i.e. nonadditive and environmental variance components) (Houle 1992; Hansen et al. 2011).

Even with recent genomic advances (most notably the ability to estimate relatedness among sampled individuals using dense genetic markers), a quantitative genetics approach to measuring evolutionary potential in natural populations has some limitations (Hansen et al. 2011; Hendry et al. 2011). By definition, measures based on additive genetic variation ignore the contributions of nonadditive effects, which manifest as dominance effects (interactions between alleles at a locus) and epistatic effects (interactions among loci) and are known to influence evolutionary trajectories (Hendry 2013). More practically, estimates of additive genetic variance require, in addition to relatedness information, an understanding of the relationship between traits and fitness (Visscher et al. 2008; Hill 2012). As genetic correlations between traits can strongly constrain evolution in response to selection, failure to account for genetic correlations will likely overestimate a population's evolutionary potential (Etterson and Shaw 2001; Hendry 2013; Munday et al. 2013). Reliably predicting a population's response to selection will thus require relatedness information for a large number of individuals, corresponding phenotypic data for traits that are correlated with fitness, an understanding of how traits relate to fitness, as well as good estimates of additive genetic variances and genetic correlations (Hill and Kirkpatrick 2010). Whilst genomic advances have enabled direct estimates of key quantitative genetic parameters (e.g. additive genetic (co)variance, genetic correlations) to be obtained for wild populations using dense genetic markers without the need for laboratory crosses or detailed non-marker-based pedigrees (Gay et al. 2013; Robinson et al. 2013), information about how traits link to fitness and ability to obtain sufficient phenotypic data will still be limited in many conservation scenarios. Estimates of evolutionary potential obtained through quantitative genetic studies will also ultimately be trait-, population- and environment-specific, restricting general application (McGuigan and Sgrò 2009; Hendry et al. 2011). Given these limitations, there is value in seeking alternative proxies for evolutionary potential in natural populations.

**Figure 1:**
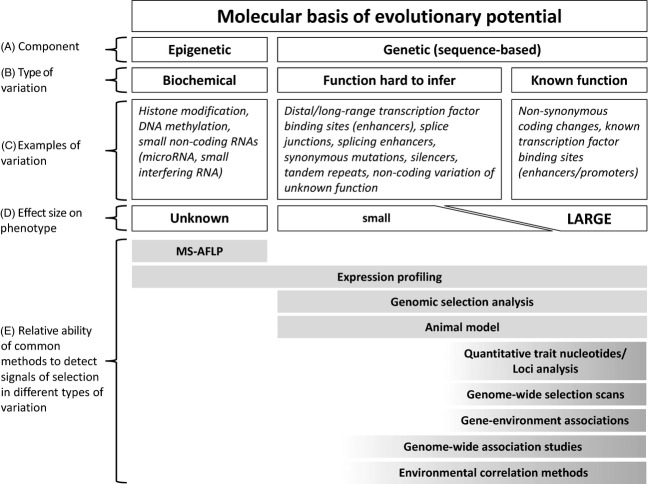
Schematic of the molecular basis of evolutionary potential. (A) The components of evolutionary potential are divided into its epigenetic (i.e. nongenetic inheritance not attributable to DNA sequence) and genetic (i.e. sequence-based) components. (B) Evolutionary potential is further divided into different types of underlying variation based on function. (C) Examples of the different types of variation are listed. (D) Different types of variation differ in their typical individual effect size on phenotype. (E) The typical ability to detect signals of selection differs between methods, as indicated by the shaded bars. Additional information about the methods in (E) can be found in Table1. Signatures of selection on epigenetic variants that are in linkage disequilibrium with sequence-based variations can be indirectly captured by all of these methods.

In addition to supplementing the application of traditional quantitative genetic approaches in some conservation scenarios (see *Using genomic advances to supplement application of traditional quantitative genetics approaches to conservation* below) (Hill 2012), genomics, in as much as it can link genetic variation to adaptively important trait variation, also provides scope for alternative measures of evolutionary potential to be developed. A robust genomic estimator of evolutionary potential would comprise weighted estimates of all adaptive or potentially adaptive genetic (including coding, regulatory and cryptic, see below), and epigenetic, variation. However, determining the genetic changes and epigenetic mechanisms underlying evolution of novel phenotypes and developmental pathways is by far the biggest challenge facing evolutionary biologists (Mackay et al. 2009; Radwan and Babik 2012). There are two major components of evolutionary potential: genetic (DNA-sequence-based) and epigenetic (non-DNA-sequenced-based; Fig.1).

### Summarizing knowledge about the genetic and epigenetic components of evolutionary potential

#### Genetic components of evolutionary potential: coding, regulatory and cryptic genetic variation

The eukaryote genome is broadly comprised of exons (coding sequence and untranslated regions, or UTRs), introns (noncoding sequence in a gene) and intergenic regions (noncoding sequence between genes). In the human genome, only ∼1.5% of sequence codes for amino acids, but around 10–15% of sequence is estimated to be functionally constrained, highlighting the evolutionary significance of noncoding sequence (Ponting and Hardison 2011). Because evolutionary approaches used to estimate functional constraint of sequence are limited by not accounting for sequences that are rapidly evolving and/or sequences that have lineage-specific functions, quantifying the amount of the genome that performs a biological function has proven difficult (Pheasant and Mattick 2007; Ponting and Hardison 2011; Mattick and Dinger 2013). Emerging genomic studies are challenging the traditional view that most of our DNA is ‘junk’ (e.g. Vernot et al. 2012; Grossman et al. 2013). Most prominently, the ENCODE Project Consortium recently assigned potential biochemical functions to ∼80% of the human genome (Dunham et al. 2012). Although the claim that most of our genome may be functional has attracted criticism, primarily concerning ENCODE's definition of a functional element (e.g. Doolittle 2013; Graur et al. 2013), ENCODE's findings have served to highlight how much there is still to learn about how genomes function and the mechanisms underlying evolution. As we still do not have a good understanding of how much of the genome is functional, it is important to remain open-minded (Ball 2013; Mattick and Dinger 2013; Mudge et al. 2013). Even if not currently functional, much of the genome is still likely to represent a pool of variation that may be brought into use in the future, further blurring the boundaries of ‘function’ (Khaitovich et al. 2006; Wagner 2011, 2012).

Despite constituting only a small fraction of most genomes, genes and their adjacent regulatory regions have been the usual focus of studies of adaptive variation under the rationale that they are the regions of discernible function. Because nonsynonymous mutations in coding regions of genes alter the amino acid sequence of a protein or a nucleotide sequence of the mature RNA, they can alter a gene product or even eliminate its function (Stern and Orgogozo 2009). Many genetic studies link specific nonsynonymous mutations (which are usually alleles of relatively large phenotypic effect) to changes in phenotypes. Whilst synonymous mutations by definition do not alter the primary amino acid sequence of a gene product, some perform regulatory roles including transcription enhancement, microRNA targeting and alternative splicing (Goode et al. 2010; Lin et al. 2011). Adaptive phenotypic changes can also be controlled by regulatory mutations that alter gene expression, and can be a predominant source of adaptation (Attanasio et al. 2013; Fraser 2013). Whilst regulatory elements often occur near or within their target genes (e.g. *cis-*regulatory elements in the upstream 5' untranslated region (UTR), the downstream 3'UTR, the promoter region and within the coding sequence), they can also occur at greater distances (e.g. *trans*-regulatory elements); enhancers often lie 10–100 kb from their target genes (Lindblad-Toh et al. 2011; Attanasio et al. 2013).

Recent studies suggest that a large proportion of adaptively important regulatory variation may reside in noncoding regions (Gaffney and Keightley 2006; Goode et al. 2010; Lin et al. 2011; Lindblad-Toh et al. 2011). For example, comparative analyses of mouse and rat genomes revealed that evolutionarily conserved elements in noncoding sequence were three times more common than those found in coding sequence, and the majority were located in intergenic regions > 5 kb from known genes (i.e. beyond the promoter region) (Gaffney and Keightley 2006). Similarly, a comparative analysis of 29 mammalian genomes revealed that the majority of evolutionarily constrained elements occurred in intronic (29.7%) and intergenic (38.6%) regions that were not associated with known protein-coding transcripts (Lin et al. 2011; Lindblad-Toh et al. 2011). Genome-wide association studies have also highlighted the evolutionary significance of noncoding sequence, finding as much as 88% of trait-/disease-associated variants lies in noncoding sequence (Altshuler et al. 2008; Hindorff et al. 2009). Although nonsynonymous mutations may have, on average, an individually larger effect on phenotype compared with regulatory mutations, the large number of evolutionarily conserved and/or trait-associated variants occurring in intergenic and intronic regions suggests a strong and previously under-appreciated contribution of noncoding sequence to phenotypes (Boyko et al. 2008; Goode et al. 2010; Vernot et al. 2012). Indeed, recent evidence suggests that regulatory changes affecting gene expression may be more important than changes in protein-coding regions in driving rapid evolution (Jones et al. 2012; Langley et al. 2012; Vernot et al. 2012; Fraser 2013; Grossman et al. 2013).

A large amount of genetic variation that is present but invisible at the phenotypic level (i.e. cryptic variation) enables populations to adapt faster to environmental change (Le Rouzic and Carlborg 2008; Wagner 2011, 2012; Masel 2013). For example, in an experiment involving *Azoarcus* derived RNA enzymes (ribozymes), populations containing large amounts of cryptic variation adapted six times faster to a new chemical environment than did populations with little cryptic variation (Hayden et al. 2011). Accumulated cryptic variants can constitute a kind of genetic ‘charge’ that can be released during periods of environmental stress (Le Rouzic and Carlborg 2008; Masel 2013). The release of charge occurs via the epistatic interactions of evolutionary ‘capacitors,’ which ‘switch on’ cryptic variation under particular circumstances. The most well-known example of an evolutionary capacitor is the chaperone Hsp90 (heat-shock protein 90) which, when down-regulated (e.g. in times of stress) in many organisms (including *Arabidopsis,* zebrafish *Danio rerio* and wild *Drosophila*), releases a variety of different phenotypes that are not expressed under benign conditions (Chen and Wagner 2012; Masel 2013). Additional examples in the literature are rare, but a recent study presented evidence for multiple capacitors that reveal subtle quantitative variation in wing morphology in *Drosophila melanogaster* (Takahashi 2013). Although there is still much to be learned, release of cryptic standing genetic variation may constitute a routine mechanism for adaptation (Masel 2013; Siegal 2013; Trotter et al. 2013).

#### Epigenetic components of evolutionary potential

Environmentally induced, transmissible phenotypic variation has been empirically demonstrated to arise from a diversity of non-Mendelian inheritance mechanisms known as ‘nongenetic inheritance’ (reviewed in Bonduriansky et al. 2012). This phenomenon comprises all parent–offspring inheritance other than DNA sequence variants and can be classed broadly under ‘epigenetics’ to include any nongenetic mechanisms (somatic, behavioural and cultural inheritance), or DNA proximate molecular-level mechanisms *sensu stricto* (Bonduriansky et al. 2012; Ledón-Rettig 2013). Here, we focus on molecular-level epigenetic factors that regulate gene expression via biochemical alterations to chromatin structure (e.g. DNA methylation and post-translational histone modifications), or through the actions of small noncoding RNAs (Ledón-Rettig 2013; Schrey et al. 2013).

Once thought to manifest only in transient, exquisite, developmental gene expression programmes that were reset in the germ line, the discovery of transgenerational stability of novel epigenetic states showed that natural variation can exist outside the DNA sequence and cause heritable variation in phenotypes even in the absence of genetic variation (Richards et al. 2010; Ledón-Rettig 2013). Viewed as an extension of conventional within-generation phenotypic plasticity, nongenetic inheritance may serve to buffer a population against rapid environmental change (Bonduriansky et al. 2012; Ledón-Rettig 2013). Some types of epigenetic change may also promote the eventual transition of adaptive mechanisms from nongenetic alterations to changes in DNA sequence (Flores et al. 2013). For example, DNA methylation is known to increase the likelihood of cytosine-to-thymine transitions and thus may increase the likelihood of sequence mutations in regions that are methylated repeatedly across generations (Flores et al. 2013). Although lack of empirical data fuels debate regarding long-term evolutionary trajectories, transgenerational epigenetic effects have added another layer of complexity to our understanding of at least short-term adaptive responses to environmental change (Bonduriansky et al. 2012; Ledón-Rettig 2013).

DNA methylation has been the most commonly studied epigenetic mechanism (Schrey et al. 2013). The technique of methylation-sensitive amplified fragment length polymorphism (MS-AFLP) is currently the most tractable option for identifying and characterizing environmentally dependent epigenetic variation in natural populations (Schrey et al. 2013; Table1). Despite some conceptual and technical limitations (reviewed in Schrey et al. 2013), there is potential for MS-AFLP, or alternative methods (e.g. bisulphite sequencing: Cokus et al. 2008; or array-based methods: Marinković et al. 2012), to be used to characterize epigenetic variation in natural populations and thus identify populations or individuals that have the greatest capacity to rapidly adjust to environment change, at least in the short-term (Liebl et al. 2013; Schrey et al. 2013).

**Table 1:** Examples and descriptions of genetic and genomic approaches commonly used in population genetics.

Approach	Description	Reference
*Mapping genes associated with traits*		
Quantitative trait nucleotides/loci programs	Use experimental crosses to look for physical location of regions of genome underlying complex phenotypic traits.	(Barton and Keightley 2002)
*Identifying loci putatively under selection*		
Genome-wide selection scans (GWSS)	Look for regions of the genome where genetic variation between populations differs relative to the genome-wide average (e.g. *F*_ST_-outliers)	(Oleksyk et al. 2010)
*Associating genetic variation with selective pressures*		
Genome-wide association studies (GWAS)	Look for associations between genetic variants and particular phenotypic traits	(Stranger et al. 2011)
Genetic–environment associations (GEA)	Look for associations between candidate loci (e.g. outliers identified using GWSS) and environmental variables	(Bierne et al. 2011)
Environmental correlation methods	Look for correlations between allele frequencies and environmental variables. Some methods control for population structure.	(Joost et al. 2007; Coop et al. 2010; Eckert et al. 2010; Hancock et al. 2010b)
*Directly identifying the genes involved in adaptation*		
Expression profiling	Looks for differential expression of genes under different conditions	(Harrison et al. 2012; Smith et al. 2013)
*Estimating additive genetic variance and genetic correlations and predicting phenotypes without knowledge of underlying genotypes*		
Animal model	Employed in animal/plant breeding. Uses sparse or dense genome-wide markers to estimate additive genetic variance and genetic correlations and to predict breeding value for phenotypes without knowing particular loci underlying traits.	(Wilson et al. 2010)
Genome-selection	Employed in animal/plant breeding. Uses dense genome-wide markers to estimate additive genetic variance and genetic correlations and to predict breeding value for phenotypes without knowing particular loci underlying traits. Requires a reference population.	(Meuwissen et al. 2013)
*Characterizing genome-wide methylation patterns*		
Methylation-sensitive amplified fragment length polymorphism (MS-AFLP)	Detects variation in methylation at restriction sites (loci) using methylation-sensitive enzymes.	(Schrey et al. 2013)

The burgeoning field of ecological epigenetics aims to characterize evolutionary and ecologically important phenotypic variation from nongenetic sources, particularly under rapid environmental change (Bonduriansky et al. 2012; Ledón-Rettig 2013; Schrey et al. 2013). Several studies have examined epigenetic effects in response to environment and as a mechanism for coping with low levels of genetic variation (e.g. Herrera et al. 2012; Richards et al. 2012). For example, in a study examining patterns of DNA methylation across an expanding population of the introduced house sparrow *Passer domesticus* in Kenya, Liebl et al. (2013) showed that epigenetic mechanisms may increase the flexibility of populations to adapt to changing environmental conditions. Epigenetic diversity was negatively correlated with genetic diversity and positively correlated with inbreeding, suggesting DNA methylation could act as a compensatory mechanism for low standing genetic variation between individuals in recently invaded populations. Limited empirical data concerning the implications of nongenetic inheritance for evolutionary potential currently constrain the general application of epigenetics to conservation. However, there is clearly scope for gathering epigenetic information alongside genetic data to understand better the relative contributions of epigenetic and genetic mechanisms to evolutionary potential in wild populations (Bonduriansky et al. 2012; Ledón-Rettig 2013).

Exploring epigenetic mechanisms can also lead to insights into other important facets of conservation. For example, devil facial tumour disease (DFTD) is a contagious cancer that has devastated Tasmanian devil *Sarcophilus harrisii* populations over the last two decades (Lane et al. 2012). An important part of the vertebrate immune response to infection involves signals from the major histocompatibility complex (MHC) that are expressed on the surface of cells and allow the host to identify ‘nonself’ cells that should be destroyed (Bernatchez and Landry 2003). DFTD evades the devil's immune system by down-regulating MHC expression on the surface of the tumour cells, a mechanism that appears to be controlled by epigenetic, rather than structural modifications (Siddle et al. 2013). Understanding the epigenetic mechanism underlying DFTD has promising implications, for example DFTD cells could be epigenetically modified to up-regulate expression of MHC in the tumour cells and subsequently used as a vaccine that would prevent the disease from evading the devil's immune response (Siddle et al. 2013).

### Using genomics to characterize adaptively important variation in the presence of polygenic adaptation

It is now understood that many – if not most – complex phenotypic traits are polygenic: they are controlled by a large number of interacting alleles of individual small phenotypic effect (Pritchard et al. 2010; Le Corre and Kremer 2012; Rockman 2012; Travisano and Shaw 2013). A growing number of studies in humans (Hancock et al. 2010a,b; Tennessen and Akey 2011; Turchin et al. 2012; Daub et al. 2013; Fraser 2013; Yang et al. 2013) and model organisms (e.g. *Arabidopsis*: Atwell et al. 2010; Lee and Mitchell-Olds 2012; *Drosophila*: Burke et al. 2010; Langley et al. 2012; Mouse: Fraser et al. 2011; Yeast: Fraser et al. 2012) have demonstrated the prevalence of polygenic adaptation. Despite this, the majority of commonly used methods for detecting loci putatively under selection and/or linking genotypes to phenotypes or environmental pressures (summarized in Table1) are strongly biased to alleles of individual large phenotypic effect (Rockman 2012; Travisano and Shaw 2013) (Fig.1).

Polygenic models of adaptation have been under-represented in population genetics, a field that has tended to favour classical sweep models focussing on shifts towards fixation of beneficial alleles at one or a few loci (Pritchard et al. 2010). Under the polygenic model, short-term adaptation from standing variation occurs via modest shifts in allele frequencies at a large number (100s) of loci (Barton and Keightley 2002; Pritchard et al. 2010). Selection across many loci in response to an environmental change produces a shift towards a new phenotypic optimum, but once the new optimum is reached, selective pressures weaken before alleles are driven to fixation (Chevin and Hospital 2008; Pritchard et al. 2010). Importantly, where large numbers (>20) of small-effect loci control a given trait, large phenotypic differences at adaptive traits can exist between populations without strong underlying differences in allele frequencies (Le Corre and Kremer 2003, 2012). Detecting signatures of polygenic adaptation using single-locus methods becomes increasingly challenging as the number of loci involved increases, particularly in situations where populations are connected by medium to high levels of gene flow and selection is recent or ongoing (Le Corre and Kremer 2012). Such scenarios are likely to exist for many natural populations, indicated by emerging evidence of polygenic adaptation in wild populations that was undetectable using standard, single-locus methods (e.g. for trees: Ma et al. 2010; Eckert et al. 2013; fish: Perrier et al. 2013; and birds: Robinson et al. 2013; Santure et al. 2013).

Genome-wide-association studies (GWASs) have revealed many novel variants associated with complex traits, but have not proven a panacea; significantly associated variants are collectively still unable to explain a substantial part of heritable variation in complex traits (Hancock et al. 2010a; Stranger et al. 2011; Rockman 2012). Although limited in their ability to detect the alleles of smallest effect (Le Corre and Kremer 2012; Rockman 2012), GWASs and environmental correlation methods can reveal subtle patterns of adaptive divergence that are too weak to be detected using standard genomic methods such as genome-wide selection scans (GWSS) or QTN/QTL programs (Joost et al. 2007; Coop et al. 2010; Hancock et al. 2010a,b; Eckert et al. 2010; Stranger et al. 2011; Fig.1). For example, Hancock et al. (2010b) inferred rapid adaptation across human populations in response to climate, diet and mode of subsistence (e.g. agriculture, foraging) based on subtle shifts in allele frequencies detected using novel Bayesian environmental correlation methods that also account for population structure (Coop et al. 2010).

Most current methods are fundamentally limited in their ability to detect polygenic adaptation because they look for signatures of selection at individual loci, rather than the combined contributions of multiple loci (Pritchard et al. 2010; Le Corre and Kremer 2012). Indeed, a large proportion of the ‘missing heritability’ that has plagued GWASs of human complex traits can be ‘found’ by considering all screened SNPs, rather than just those with significant phenotypic associations (Yang et al. 2010, 2013). For example, Yang et al. (2010) increased the explained amount of phenotypic variance in human height from 5% to 45% by considering all SNPs simultaneously, rather than just focusing on the large-effect SNPs that pass stringent significance testing. Yang et al. (2013) further demonstrated the ubiquity of polygenic adaptation, finding that for 49 human complex traits, an average of one third (range: 8–77%) of phenotypic variance could be explained using >300 000 SNPs on a particular genotyping array. The majority of remaining unexplained variance was likely due to causal variants not included in the SNP array: any ∼1% of the genome explained ∼1% of the heritability, with the implication that whole-genome sequencing data (as opposed to SNP array data) should explain all phenotypic variation in the trait (Yang et al. 2013). Novel methods are emerging that look for coordinated shifts in allele frequencies across sets of loci, rather than at individual loci (Turchin et al. 2012; Berg and Coop 2013; Daub et al. 2013; Fraser 2013). For example, alleles known *a priori* to be associated with increased height were systematically higher in frequency in northern than in southern European populations, providing one of the first empirical examples of widespread weak selection acting on standing genetic variation (i.e. polygenic adaptation) in humans (Turchin et al. 2012). An approach that jointly considers all genes involved in a given biological pathway and tests them for signatures of positive selection has also proved a novel and powerful method for detecting polygenic adaptation (e.g. Daub et al. 2013; Fraser 2013).

Although studies considering the combined contributions of multiple loci are rare in nonmodel systems, pioneering examples do exist. For example, Ma et al. (2010) sought signatures of selection across populations of European aspen *Populus tremula*, finding that 20–25% of phenotypic variation in growth cessation (a trait involved in adaptation to different light regimes) could not be attributed to individual SNPs, but could be explained by positive covariance in allelic effects. Similarly, for another well-studied tree species, the loblolly pine *Pinus taeda*, Eckert et al. (2013) found that for many phenotypic traits, signatures of selection were evident only when loci were considered at the level of functional sets (i.e. across all loci associated with a particular trait). The methods used in both these examples require some prior knowledge of loci underlying traits of interest (e.g. obtained through genome-wide-association studies) and thus may not be currently feasible for many species of conservation concern, for which resources may be limiting. This may change, however, as human genetics advances and new methods for detecting polygenic adaptation are developed.

### The role of gene expression studies in conservation

Rapid adaptive evolution is driven predominantly by changes in gene expression (Jones et al. 2012; Fraser 2013) enabled by the presence of substantial variation in gene expression within natural populations (Oleksiak et al. 2002). Variation in gene expression is routinely approximated by mRNA abundances (the result of transcriptional activity of genes), although, as recently inferred from a comparison of quantitative proteomics and RNA-seq data, expression of proteins may depend primarily on the translational efficiency of specific genes (Taylor et al. 2013). Expression profiling (simultaneous measurement of RNA production by multiple genes) can be a valuable tool for assessing the combined outcomes of many subtle, complex processes affecting gene expression, including polygenic and epigenetic effects. Gene expression studies can also identify many of the genomic regions involved in phenotypic traits, providing information about molecular mechanisms and pathways involved and, in conjunction with other approaches (e.g. GWAS), enabling the identification of some of the specific underlying genetic variants (Attanasio et al. 2013; Filteau et al. 2013).

Traditionally limited to genes known from model organisms (assayed via qPCR analyses of candidate genes or cross-species hybridization on microarrays), gene expression studies were unleashed for use in nonmodel organisms by the advent of high-throughput sequencing of RNA (RNA-seq). Many such studies begin with a *de novo* assembly of a transcriptome (a collection of RNA molecules produced by a tissue that are either directly translated to proteins or are apex gene products themselves) (Schliesky et al. 2012) followed by annotation of exons using the closest available reference genomes (Vijay et al. 2013). Variation in gene expression of large numbers of individuals is then assessed either directly by RNA-seq and subsequent quantification of reads mapped back to the assembled transcriptome (Smith et al. 2013) or by designing a custom microarray to quantify the expression of only a subset of genes contained in transcriptome (Renaut and Bernatchez 2011; Kvist et al. 2013).

Expression of many genes is highly tissue-specific (Ekblom et al. 2010a) and can be influenced by environmental conditions (Regier et al. 2013), previous experience of the organism (Feil and Fraga 2012), life-stage (Arbeitman et al. 2002) and sex (Vidotto et al. 2013). Tissue- and environment-specificity currently limit gene expression studies to situations where experimental manipulations in controlled laboratory environment are feasible and animals can be sacrificed for tissues. This makes application of gene expression studies in animal conservation challenging (e.g. only some genes, mainly of general function, would be expressed in blood or small skin tissue samples, the kind regularly obtained by conservation studies from live animals; Ekblom et al. 2010b). Nevertheless, characterization of transcriptomes will continue to be a valuable method in conservation for the detection of candidate loci for future targeted screening (e.g. Renaut et al. 2010; Bi et al. 2012).

Several challenges are associated with the analysis of gene expression data (reviewed in Harrison et al. 2012). These include (i) accounting for nonadditive effects of mutations, direction of expression change, gene copy number variation, alternative mechanisms of expression level regulation and environmental effects, and (ii) distinguishing nonadaptive differences in gene expression that are due to genetic drift from adaptive differences that are due to positive selection for advantageous traits (Harrison et al. 2012). Addressing these challenges will involve development of (i) a robust model of evolution of gene expression and (ii) an appropriate (and possibly tissue-specific) null model of neutral evolution of gene expression (Harrison et al. 2012). To understand better the role of adaptation and plasticity (genotype-environment effect) in the evolution of highly polygenic traits, it is important to explore gene expression variation from a network perspective (Filteau et al. 2013), for example, using weighted gene co-expression network approach (WGCNA; Oldham et al. 2006) or the approach developed by Fraser (2013).

Notwithstanding these challenges, gene expression studies published in the past few years have made a substantial contribution to our understanding of evolution, including insights into adaptive responses to environmental stressors (crimson spotted rainbowfish: Smith et al. 2013; the rooted macrophyte *Elodea nuttallii:* Regier et al. 2013), the evolution of life-history traits and life-history trade-offs (Glanville fritillary butterfly *Melitaea cinxia*: Wheat et al. 2011; Kvist et al. 2013), immunological adaptations (three-spined sticklebacks *Gasterosteus aculeatus*: Lenz et al. 2013), reproductive isolation and ecological speciation (reviewed in Pavey et al. 2010) and adaptive radiation (cichlid fish: Manousaki et al. 2013). Genes differentially expressed under different environmental conditions could be involved in adaptation to a particular environmental factor and thus are of interest to conservation projects concerned about adaptations to future environments (Harrison et al. 2012; Smith et al. 2013). A combination of genomic approaches applied to ecological model systems of evolutionarily young and ecologically distinct lineages of nonmodel organisms (e.g. Bernatchez et al. 2010; Jones et al. 2012) will continue to lead to important insights into the mechanisms underlying adaptive evolution.

### Estimating evolutionary potential from genomic information in a management-dependent context

Earlier we presented evidence that adaptive variation resides in both coding and noncoding sequence and is predominantly polygenic and regulatory in nature. Further, gene expression can be strongly altered by non-sequence-based epigenetic variation, and even variation invisible at the level of the phenotype (i.e. cryptic variation), or not currently adaptive, may still contribute to a population's evolutionary potential. Much of this complexity is difficult to detect using standard population genomic methods, and limitations of current methods need to be considered when designing the screening approach, and when basing management decisions on genomic data (e.g. by screening coding and noncoding parts of the genome and not routinely biasing management decisions to large-effect loci). Embracing recent advances in other fields (e.g. quantitative genetics, human genetics, epigenetics, expression profiling) and factoring in the contributions of polygenic adaptation, as well as cryptic and epigenetic variation, should result in better-informed applications of conservation genomics and better estimates of evolutionary potential than have been previously possible for most wild organisms.

**Figure 2:**
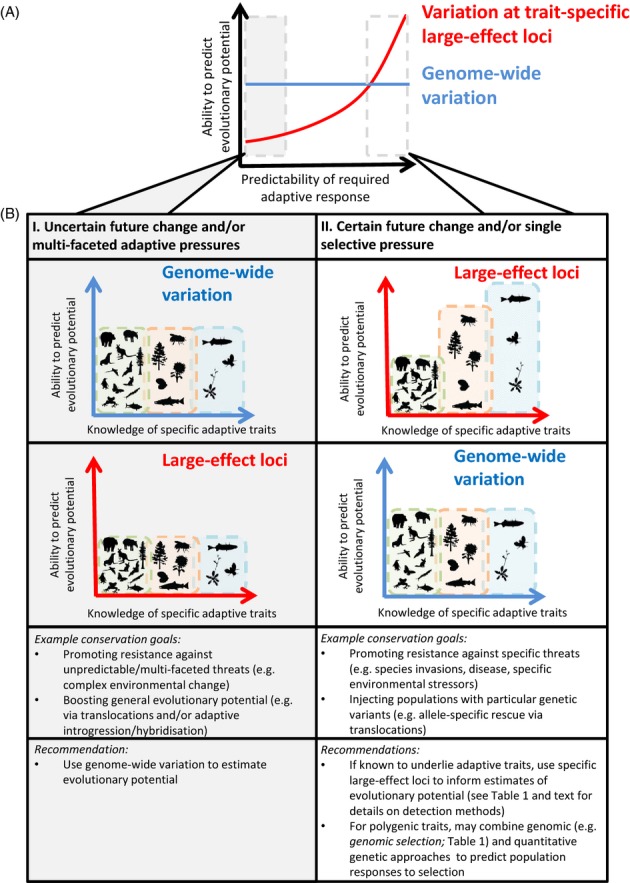
Schematic exploring the relative abilities of two broad genomic approaches to predict evolutionary potential, given specific trait architectures, environmental circumstances and levels of prior genetic knowledge. The two broad approaches (screening genome-wide variation and screening variation at specific large-effect loci) should not be treated as mutually exclusive alternatives, as they can be complementary (e.g. screening genome-wide variation in conjunction with specific loci of interest). For the case of organisms where there is good genetic knowledge available, (A) shows the relative ability to predict evolutionary potential based on variation at trait-specific large-effect loci (red) versus genome-wide variation (blue), with increasing ability to predict the specific required adaptive response. (B) Takes the two extremes of (A) and considers relative ability to predict evolutionary potential for model organisms (blue shaded bar), commercially valuable species (orange shaded bar) and typical species of conservation concern (green shaded bar) under I) uncertain future change and/or multi-facetted adaptive pressures (left panel) and II) under certain future change and/or single selective pressures (right panel). The blue axes plots correspond to relative ability to predict evolutionary potential using variation at trait-specific large-effect loci and the red axes plots correspond to relative ability to predict evolutionary potential using genome-wide variation. Recommendations for when the two different approaches should be employed and examples of the types of conservation goals that could be addressed using the two different approaches are given.

As we are unlikely to know in advance the best course of conservation action, a risk-management approach is appropriate for making initial decisions (*sensu* Frankham et al. 2011; Weeks et al. 2011), with subsequent actions subject to an adaptive management framework (i.e. choose the apparent best path, monitor the outcomes, then adjust the path according to outcomes) (Hansen et al. 2012). Whilst we assemble knowledge and explore the effectiveness of different genomic (and epigenomic) measures of evolutionary potential, deciding what is appropriate for a given project will depend on how well genomic control of relevant adaptive traits is understood, the level of certainty in predicted selective pressures and the genetic architecture of the trait(s) involved (i.e. underlying large-effect or small-effect loci). Below we discuss two broad, complementary genomic approaches that can be employed to greater or lesser extents, depending on the particular management scenario (summarized in Fig.2). Here, management scenario encompasses the knowledge of genomics and adaptation of species, predictability of required adaptive response, genetic architecture of the trait(s) involved and specific conservation goal, all within the context of promoting evolutionary potential in wild populations. Our intention is to highlight conservation scenarios in which it may be appropriate to focus on specific, trait-based information and when it might be preferable to focus on genome-wide variation.

#### Utilizing specific, trait-based information to inform estimates of evolutionary potential

When high-quality genetic information is available in combination with good predictions about the nature of future environmental change, genetic variation associated with relevant traits controlled by a small number of genes may be informative about evolutionary potential (e.g. potential to adapt in response to known selective pressure). There are well-known examples of traits known to be controlled by a small number of large-effect QTLs, including armour plating associated with marine-freshwater divergence in sticklebacks, (Albert et al. 2008), and flowering time in *Arabidopsis thaliana* (Salomé et al. 2011).

Including *a priori* identified candidate genes in genomic screens can be beneficial because something is already known about the kinds of adaptive responses the genes are involved in (e.g. ‘bottom-up’ approach *sensu* Sork et al. 2013). Genes predicted to be adaptively important can be selected using prior knowledge available and/or additional complementary approaches (e.g. expression profiling). As coding regions are typically relatively conserved, candidate genes can often be selected using the closest reference genome (Vijay et al. 2013). Alternatively, loci putatively associated with particular kinds of adaptation can be identified *post hoc* from analyses of genomic sequence data (e.g. ‘top-down’ approach *sensu* Sork et al. 2013) (Table1).

Under a scenario of divergent selection between two large populations that remain connected by moderate to high levels of gene flow (necessary to homogenize the neutral genomic background), anonymous large-effect loci associated with the adaptive divergence can be detected using standard genome-scan methods (Le Corre and Kremer 2003, 2012). Such scenarios do exist in nature; for example, whole-genome sequencing of three-spine sticklebacks from replicated pairs of freshwater and marine populations (connected by ongoing gene flow) identified 242 regions that were repeatedly associated with marine-freshwater divergence (Jones et al. 2012). If it was predicted that a marine stickleback population would be required to adapt to freshwater conditions under environmental change, then considering variation at the 242 regions associated with freshwater adaptation (or ideally a validated subset of loci) may be informative about that population's evolutionary response.

In specific conservation situations analogous to the case just described, it might be appropriate to focus management on specific types of diversity needed to adapt to a current major threat posing immediate extinction risk to the organism. Identification of immunologically important variation (e.g. associated with the major histocompatibility complex in animals) that provides resistance to disease epizootics in the wild could be used to inform selection of resistant or immune individuals for captive breeding, reintroductions and other forms of population genetic augmentation. Key examples might include resistance to chytrid fungus in frogs (Savage and Zamudio 2011) and facial tumour disease in Tasmanian devils (Lane et al. 2012). Additional candidates for allele-specific genetic rescue (*sensu* Allendorf et al. 2010) might include self- incompatibility loci in plants (Hoebee et al. 2008), *Pgi* in insects (linked to energy metabolism; Wheat et al. 2011), *Pan*I in the cod family (linked to temperature, salinity and water depth; Árnason et al. 2009) and, more generally, heat-shock protein genes related to temperature stress (Sørenson et al. 2003) and circadian genes related to phenological traits (Liedvogel et al. 2009; Jimenez et al. 2010). Although prioritization of specific adaptive variants in conservation management decisions (e.g. selecting individuals for translocations in a way that enriches for genotypes predicted to be adaptively important in the recipient population) may have advantages, it risks depleting populations of other sources of adaptive variation unless measures of broader genome-wide diversity are also considered.

The potential to use knowledge about the genetics of specific traits to predict evolutionary potential will be greatest when the traits are controlled by a small number of genes. However, the ability to predict a population's evolutionary potential will ultimately hinge on having a reasonable degree of certainty in the predicted consequences of environmental change and an ability to predict with confidence the specific adaptive variant(s) that will be required. An extreme illustration is provided by toxic invasive cane toads (*Bufo marinus*), which cause almost certain, rapid death in susceptible individuals, leading to massive population declines and local extinctions (Doody et al. 2009). Extremely effective biochemical resistance is provided by a few amino acid changes in one domain of one protein (Ujvari et al. 2013). In the absence of other viable management options, and given the predictable geographical progress of the invader, evolutionary rescue via resistance genes could be planned with some confidence. Despite rare examples such as cane toad invasion, uncertainty about required adaptive response(s) is likely to be much more commonplace, especially because knowledge of what is adaptive today will not necessarily translate into knowledge about what will be important tomorrow (Le Corre and Kremer 2003; Allendorf et al. 2010).

#### Utilizing genome-wide variation to inform estimates of evolutionary potential

In most conservation situations, genomic knowledge about traits (loci involved, additive genetic (co)variances, genetic correlations and genetic architecture) will be limited, and/or the predictability of environmental change and the required adaptive response(s) will be low. Under this scenario, screening genome-wide variation should be a sensible approach that may provide a generalized measure of evolutionary potential that accounts for the contributions of small-effect loci and cryptic variation and is robust to uncertainty about future change and required adaptive response(s). We use the term ‘genome-wide variation’ in a broad sense to reflect variation sampled representatively across the genome, which could encompass a range of metrics, including allelic diversity, heterozygosity and weighted measures that place greater importance on specific loci of interest within the context of genome-wide variation.

A possible drawback of using genome-wide estimates of evolutionary potential is the inclusion of genetic variation that might be currently nonfunctional, and hence not necessarily informative about evolutionary potential. However, two main lines of evidence support genome-wide measures of variation as appropriate proxies for evolutionary potential. First, limited data from simulation (Caballero and García-Dorado 2013) and empirical (Coop et al. 2009) studies suggest that patterns at a large number of random or neutral loci are likely to be correlated with variation at a smaller number of QTL or *F*_ST_-outlier loci. Second, and more significantly, for highly polygenic traits, genome-wide variation currently provides the best predictions of phenotypes (Meuwissen et al. 2013; Yang et al. 2013).

Screening genetic variation at a large number of genome-wide markers is a method already extensively used in animal and plant breeding to predict phenotypes without specific knowledge of the contributing loci (Meuwissen et al. 2013). This method (called genomic selection) uses information from all genotyped loci (typically ≫ 10 000 SNPs) to predict breeding values for desired phenotypic traits that may not be evident before an animal/plant reaches minimum breeding age (Meuwissen et al. 2013; Table1). Trait value predictions are achieved through comparison with a training population that has been measured for the trait of interest and genotyped using the same set of markers. By using all loci to predict phenotypes, genomic selection can also account for the contributions of small-effect alleles (Meuwissen et al. 2013; Fig.1).

### Scope for genomic advances to supplement application of traditional quantitative genetics approaches to conservation

In some conservation situations (akin to panel II in Fig.2B), genomic advances may feed well into a quantitative genetics framework and allow for predictions of evolutionary potential and population responses to selection. As we outlined earlier, by supplying relatedness information, dense marker genotyping afforded by genomic advances has made previously unfeasible estimation of key quantitative genetic parameters (e.g. additive genetic (co)variance, genetic correlations) possible for nonmodel populations (Gay et al. 2013; Robinson et al. 2013). Marker-based animal models and genomic selection methods are two promising, broad approaches for the estimation of additive genetic variance and genetic correlations in natural populations (Wilson et al. 2010; Sillanpää 2011; Hill 2012; Gay et al. 2013; Table1). Such multimarker methods have been used to estimate the additive genetic variance of traits in humans (Yang et al. 2010, 2011; Stanton-Geddes et al. 2013) and in wild bird populations (wing length: Robinson et al. 2013). In a pioneering study, Robinson et al. (2013) used thousands of genome-wide SNPs to construct a pairwise relatedness matrix among individuals from an ecological study population of great tits *Parus major*. Estimates of additive genetic variance for a complex quantitative trait (wing length) were derived using the relatedness matrix and then validated using extensive simulations and known pedigree information.

Notwithstanding limitations discussed earlier, the prospect that coancestries (i.e. genetic relationships among individuals), additive genetic (co)variances and genetic correlations could be estimated in natural populations using large-scale genomic data without the need for detailed pedigrees is an exciting one and would open up a raft of possibilities for exploring trait architecture and evolutionary potential in nonmodel systems (de Cara et al. 2013; Edwards 2013). In quantitative genetics, a population's response to selection at a single trait (i.e. how the mean value of a trait changes across generations in response to selection) is measured using the breeder's equation: *R* = *h*^2^*S*, where *R* is the per generation response to selection, *h*^2^ is the heritability and S is the selection differential (Lynch and Walsh 1998). A population's response to selection will thus hinge on both the intensity of selection and on the additive genetic variance associated with the trait of interest. Because in natural systems, selection is likely to act on a set of traits simultaneously, genetic correlations between traits must be accounted for using a multivariate version of the breeder's equation: *R* = *G*β, where *G* is the additive genetic covariance matrix and β is the selection gradient (Lande and Arnold 1983; Hill and Kirkpatrick 2010). Although the breeder's equation has proven a poor predictor of phenotypic trends in wild populations (many explanations are given including poor estimates of additive (co)variances and selection, failure to measure all correlated traits, changing environments), genomics advances may lead to some improvements (Kruuk et al. 2008). Genomics has enabled estimates of additive genetic (co)variance and genetic correlations, although selection intensities must still be obtained through estimates of the association between phenotypes and relative fitness (or breeding values *sensu* animal and plant breeding), which may often be challenging in wild populations (Lynch and Walsh 1998; Visscher et al. 2008). Nonetheless, in conservation situations where the required adaptive response is predictable and phenotypic data for relevant traits and fitness information are attainable, genomic estimates of additive genetic variance and genetic correlations for sets of adaptively important traits could be integrated into a quantitative genetics framework and used to predict a population's response to a particular selective pressure.

### Using genomic models to inform natural systems and the need for empirical studies that link genomic estimates of evolutionary potential to population persistence

Although infeasible for most wild populations, integrating genomic approaches into an experimental evolution framework (Evolve and Resequence/E&R *sensu* Turner et al. 2011) is a useful approach for elucidating adaptation architecture (Kawecki et al. 2012; Tobler et al. 2014). E&R applied to model systems may yield information about the genetic basis of adaptive traits that in some circumstances may be transferable to related taxa and natural systems (Kristensen et al. 2007). For example, Li et al. (2013) simulated future climate change scenarios in growth chambers and used GWAS to look for corresponding shifts in the genetic architecture of flowering time in *Arabidopsis thaliana* (a trait known to be controlled by a relatively small number of large-effect QTL). The authors were able to identify major QTL influencing the thermal sensitivity of flowering time and to build a genetic model that was able to successfully predict flowering time of given genotypes in their future climate scenarios. Approaches that link genotypes to their environments within an experimental evolution framework can identify the loci that may promote adaptive responses to environmental change. Although there are obvious limitations to applying these experimental approaches directly to many wild populations, there is good scope for application to commercially valuable crop and animal species and potential for some information to transfer to related wild taxa (Li et al. 2013).

Pragmatic conservation does not necessarily require particular phenotypes to be linked to their respective genotypes. Rather, pragmatic conservation requires sequence-based estimates of evolutionary potential to be linked to the likelihood of persistence (i.e. population viability) under future environments (Allendorf et al. 2010). Understanding how estimates of evolutionary potential translate into population viability is crucial if population viability analyses (PVAs) are to assist management decisions effectively. Although genomics has the potential to provide more representative estimates of evolutionary potential than have been previously feasible, the nature and extent of relationships between genome-wide diversity, fitness and population viability needs to be established (Allendorf et al. 2010).

A feasible approach might be to calibrate genomic estimates of evolutionary potential in nonmodel organisms with estimates from model populations where evolution has been more rigorously measured. Past empirical studies of model organisms (e.g. *Drosophila*) have looked for correlations between traditional measures of standing genetic diversity (e.g. based on a handful of microsatellite markers) and either fitness and/or evolutionary potential in response to environmental stressors (Reed and Frankham 2001; Gilligan et al. 2005; Bijlsma and Loeschcke 2012). There is scope for these studies to be repeated using genomic sequence data to compare different kinds of measures of genome-wide variation (e.g. unweighted or weighted towards particular adaptive variation) and determine which measures can provide robust predictions of evolutionary adaptation and population persistence. If higher estimates of evolutionary potential based on particular measures of genome-wide variation consistently reflect a higher likelihood of population persistence under multiple, different environmental stressors, then uncertainties surrounding future environmental conditions become less problematic, at least in the context of key conservation management questions concerning evolutionary potential.

## Conclusions

Here, we have outlined some of the complexity regarding adaptation genetics and explored limitations and recent advances in the application of genomic tools to conservation. Recent advances in the fields of quantitative and human genetics have revealed that adaptively important variation is dispersed throughout the genome, with regulatory variants and epigenetic mechanisms playing an important role in shaping gene expression and rapid adaptive evolution. Polygenic adaptation is widespread and even cryptic genetic variation that is hidden at the phenotypic level may make an important contribution to evolutionary potential. The complexity of adaptation genetics needs to be appropriately considered when planning genomic screens and subsequently when basing management decisions on genomic data. When the genomic basis of adaptation and future threats are well understood, it may be appropriate to focus management on particular adaptive traits that are likely to be of importance. For more typical conservation situations, we argue that screening genome-wide variation may provide bet-hedging estimates of evolutionary potential that account for small-effect and cryptic variants and are relatively robust to uncertainty about future environments and required adaptive change. Mining such data to understand adaptation within an adaptive management framework of conservation actions, integrating genomic advances into a quantitative genetics framework, empirically testing the effectiveness of genomic measures of evolutionary potential relevant to population persistence, and exploration of the relative contributions of epigenetic and genetic mechanisms to evolutionary potential are exciting fields in the development of conservation genomics.

## Supporting Information

**Appendix S1** Summary of common genomic methods used for marker development and screening individuals for variation at large numbers of loci.
