# Supplementary material for: Using genomics to characterize evolutionary potential for conservation of wild populations
Source: Evol Appl. 2014 Mar 14;7(9):1008–25. doi: 10.1111/eva.12149 (PMC4231592; doi:10.1111/eva.12149)
Supplement: Supplementary file 1 — Appendix S1 Summary of common genomic methods used for marker development and screening individuals for variation at large numbers of loci. [file eva0007-1008-sd1.docx]

**Appendix S1: Summary of common genomic methods used for marker development and screening individuals for variation at large numbers of loci.**

The advent of next-generation sequencing technologies (high-throughput, massively parallel sequencing) means it is now possible to screen large numbers of individuals for large numbers (thousands to tens of thousands, as opposed to tens to hundreds) of genetic markers at relatively low cost (Cosart *et al.*, 2011, Bi *et al.*, 2012, Lemmon *et al.*, 2012). Next-generation sequencing (NGS) is replacing ‘first generation’ automated Sanger sequencing and encompasses a range of technologies including Roche/454, Solexa/Illumina, Life Technologies/SOLiD, Life Technologies/Ion Torrent, Pacific Biosciences/PacBio. We do not detail the technical aspects or relative merits of NGS technologies here, but a number of good reviews have done so elsewhere (e.g. Metzker, 2010, Zhang *et al.*, 2011).

For the aforementioned NGS platforms, with the exception of PacBio, DNA (or cDNA that has been synthesised from an RNA template in the case of transcriptome sequencing) is broken into many small fragments, to which short adapter sequences enabling amplification and sequencing are attached. Unique identifier barcode sequences can be attached to fragmented DNA from particular samples (e.g. individuals or groups of individuals). Barcoded samples (libraries) are then pooled for sequencing, allowing simultaneous processing of large numbers of individuals. The libraries are then sequenced using a NGS platform. Unique barcodes allows sequences to be sorted bioinformatically and analysed separately post-sequencing (e.g. for the purposes of individual genotyping). Millions of short reads (fragments of sequence) are generated through NGS that can be either be mapped to an existing reference genome (e.g. in the case of model organisms) or assembled into longer contiguous sequences (contigs) using overlapping regions (i.e. *de novo* assembly in organisms lacking a reference genome).

Most NGS platforms are currently limited to substantially shorter sequence read lengths (35-500 bp) than is Sanger sequencing (~1000-1200bp). Since most organisms of conservation interest are not model organisms and lack a reference genome, getting adequate coverage (i.e. a number of sequence reads containing a particular base) of the region of interest is important for getting enough overlapping regions to accurately assemble longer sequences. Because NGS comes with high sequencing error rates (variable across platforms, but up to ~1%), adequate coverage is also important for distinguishing true variation at a site from sequencing error. Mapped reads and/or assembled longer sequences can be analysed in different ways depending on the type of sequence and the question of interest. Here we focus on population genomics methods (exploring patterns of sequence variation among individuals or populations). We will not discuss methods that focus on differences in gene expression among individuals or populations, which are reviewed elsewhere (e.g. Gilad *et al.*, 2009).

*Screening individuals for variation at thousands of loci (cheaply)*

There is a range of genomic approaches for screening individuals for thousands to tens of thousands of markers (Table A1). Whole-genome sequencing of a limited number of individuals of eukaryotes is now possible and within the budgets of some research projects (e.g. twenty flycatchers in Ellegren *et al.*, 2012). However, most conservation-related studies require sample sizes in the hundreds, and so the cost would still be prohibitive with current technologies, and in any case would often represent excessive data (Grover *et al.*, 2012). Instead, many studies focus their resources on obtaining adequate depth of coverage of a subset of the genome, for a larger number of individuals. Examples of approaches to such ‘genome reduction’ are described in Table A1.

Genome reduction methods usually have substantial costs associated with 1) steps that select for the part of the genome of interest, and 2) attaching barcodes to PCR products (Mamanova *et al.*, 2010), or constructing individual barcoded or ‘indexed’ libraries (Bi *et al.*, 2012). Eventually it may become more cost-effective to perform whole-genome sequencing even if all the data are not needed. But until then screening for a subset of variation provides adequate information for most questions of interest to conservation, and is feasible for some medium- or larger project budgets (Mamanova *et al.*, 2010, Lemmon & Lemmon, 2012, Peterson *et al.*, 2012).

The costs and benefits of each of the approaches outlined in Table A1 depends on whether or not genomic information exists for the organism of interest (e.g. a reference genome for the species of interest or a closely related organism) and also on the biological question of interest. For example, shotgun sequencing can provide useful information for marker development but in most cases is not feasible as a screening technique because the depth of coverage needed for repeatable and accurate genotyping would be too costly. Targeted sequence enrichment (in particular hybridisation-based sequence capture) offers a repeatable screening technique that can be customised to regions or types of regions of interest known *a priori* in the genome or transcriptome. Other approaches such as restriction site-associated DNA sequencing (RAD-seq) offer cost-effective screening approaches that capture a representative subset of the genome without requiring any prior information, but at the cost of producing markers that are anonymous unless a reference genome is available.

Until recently, for non-model organisms, the approaches described in Table A1 have been used predominantly in marker development to identify sets of SNPs to screen, rather than being employed directly as the method of screening individuals. Screening has tended to employ various technologies to score SNPs, which have had advantages including relative affordability, high numbers of markers targeted (in the order of 10,000s-100,000s), low scoring error rates and high repeatability (Garvin *et al.*, 2010, Helyar *et al.*, 2011). However there are also a number of challenges with using SNPs, including avoiding ascertainment bias during SNP discovery study designs, and low retrievability of information regarding the linkage associations among SNPs within and among individuals (Garvin *et al.*, 2010, Helyar *et al.*, 2011). Also, costs have not fallen as sharply as envisaged previously (e.g. Morin et al. 2004), and are being undercut by sequencing-based approaches. Thus it seems likely that SNPs for many applications will give way to approaches based on longer sequences (Narum *et al.*, 2013).

Screening for polymorphism in multi-allelic sequences, as opposed to the single-base differences revealed by SNP screening, has greater power and flexibility in terms of analytical approaches (Buerkle *et al.*, 2011). One key example is that coalescent approaches based on sequences are an extremely important source of information on amount of gene flow, timing of divergence and effective population sizes of populations (Marko & Hart, 2011). Sequence-based analyses require haplotype information, which can be provided by only a subset of the approaches outlined e.g. paired-end RAD sequencing (Table A1) (Hohenlohe *et al.*, 2013).

Screening for sequence-based polymorphism is becoming feasible and cost-effective for application to non-model organisms, notably by a combination of bar-coding, multiplexing (simultaneous analysis of multiple individuals), and genome reduction by sequence capture, RAD-seq and reduced representation libraries (RRLs) (Hohenlohe *et al.*, 2010, Cosart *et al.*, 2011, Bi *et al.*, 2012, Lemmon *et al.*, 2012). For targeting of specific genes or homologous gene regions across many taxa, hybridisation-based sequence capture (array-based or in solution) is a useful approach (Table A1). It requires some prior genomic information, not necessarily from closely related species, as the basis on which to design probes (‘baits’) that can target specific regions of interest. Recent studies have demonstrated application of sequence capture to organisms lacking a reference genome through design of probes in evolutionarily conserved regions (typically exons, i.e. coding regions) based on very distant reference genomes of other taxa (Faircloth *et al.*, 2012, Lemmon *et al.*, 2012). This targeting can capture exons as well as their adjacent regulatory and non-coding regions, providing a range of markers that differ in their function and variability. These regions (in the order of 1000s) are then sequenced using NGS to find polymorphism. Designing probes in evolutionarily conserved regions allows for the same capture system to be applied to a range of taxa (subject to how conserved the probe regions are). As more reference genomes become available, it will be increasingly feasible to design capture systems specific to particular taxa or groups of taxa (e.g. Cosart *et al.*, 2011, Bi *et al.*, 2012).

Table A1. Summary of common NGS approaches used for marker development and screening individuals for variation at large numbers of loci. Table includes pros and cons relating to use to approaches in conservation projects with limited budgets.

| Technique | Summary | Pros | Cons | References |
| --- | --- | --- | --- | --- |
| Whole genome sequencing (WGS) and re-sequencing | Sequencing and assembly of entire genomes. | Most comprehensive sequencing strategy. Most viable for organisms with small genomes (e.g. prokaryotes). | Still prohibitively expensive for most conservation project budgets; amount of data generated requires a lot of data analysis and is overkill for many research questions. | (Ellegren *et al.*, 2012, Grover *et al.*, 2012, Jones *et al.*, 2012) |
| *Shotgun sequencing* | | | | |
| Whole genome shotgun sequencing (WGSS) | Genomes are fragmented into segments of random length and sequenced. Produces multiple overlapping reads that can be mapped to a reference or assembled *de novo.* | Comprehensive sequencing strategy (although at low coverage will obtain only a random subset of genome). | Limited as a screening approach because not repeatable; difficult to assemble without a reference unless high coverage (cost prohibitive). Some representation biases. | (Altshuler *et al.*, 2010, Birol *et al.*, 2013) |
| Whole transcriptome shotgun sequencing (WTSS) or RNA-sequencing (RNA-seq) | Sequencing the transcriptome (expressed regions of the genome or exome). mRNA is fragmented then converted to cDNA using reverse transcription. Adapters/barcodes are incorporated with cDNA fragments. Fragments are size selected then amplified and sequenced  [Note RNA-seq experiments are most often used to look for differentially expressed genes among samples] | Captures functionally important variation; since proportion of coding DNA is only ~2-5% in most vertebrates sequencing, transcriptome will produce much greater coverage than WGS with equivalent sampling effort. | Challenging without a reference genome; difficulties associated with splice variants; captures only one type of variation (i.e. expressed variation at the time of sampling in the sampled tissue, which must be extremely fresh to obtain RNA). Sampling is usually lethal. | (Everett *et al.*, 2011, Helyar *et al.*, 2012, Limborg *et al.*, 2012) |
| *Targeted sequence enrichment (can target genome or exome or both)* | | | | |
| Hybridisation-based sequence capture (array or in solution) | Targeted regions are chosen based on prior information and target-specific probes designed. Sample DNA is fragmented and adapters/barcodes are incorporated. Then DNA fragments are hybridised to target-specific probes (either located on an array surface or in solution). Only the targeted DNA regions are sequenced. | Best targeted sequence enrichment strategy when many samples and targets; same array can be applied to related taxa; flexibility to build a customised set of target baits; repeatable as a screening technique. | Requires *a priori* genomic information for identifying targets/probe design. Efficiency of capture is typically reduced if target taxa are >5% diverged from reference taxa for which baits were designed (putting probes in ultra-conserved regions can increase efficiency but potentially decrease captured genetic variation). | (Mamanova *et al.*, 2010, Cosart *et al.*, 2011, Bi *et al.*, 2012, Faircloth *et al.*, 2012, Lemmon *et al.*, 2012) |
| PCR-based amplification | Uniplex (1 reaction = 1 amplified product) PCRs were used extensively for Sanger sequencing. Multiplex (1 reaction = multiple products). After PCR, concentration of products (amplicons) must be normalised. Amplicons are barcoded, pooled and sequenced. RainStorm platform can target up to 20,000 markers in a single reaction using microdroplet technology (eliminating the normalisation step) | Repeatable as a screening technique. | Multiplexing is complicated and expensive, requires *a priori* genomic information for identification of target regions and primer design, Difficult to design a multiplex that would result in equimolar PCR products across loci. | (Mamanova *et al.*, 2010) [ThunderStorm™ System](http://raindancetech.com/targeted-dna-sequencing/thunderstorm/) (http://raindancetech.com/targeted-dna-sequencing/thunderstorm/) |
| Molecular inversion probe-based amplification | Single-stranded probes hybridise to complementary target region. The ends of the probe are complementary to either end of the target region. DNA polymerase extends copies the target and fills the gap between the probe ends creating a circular DNA. Circularised regions are amplified and sequenced. | Most relevant for projects with small numbers of target regions but lots of samples. | Capture uniformity is low (issue in terms of repeatability) | (Mamanova *et al.*, 2010, Metzker, 2010) |
| *Reduced representation sequencing* | | | | |
| Reduced representation libraries (RRLs) | DNA is fragmented using restriction enzymes. Fragments from all samples are pooled (i.e. no bar-coding of individuals) and then size-selected. Standard sequencing adapters are attached to the ends of remaining fragments and fragments are sequenced. | Obtain random, genome-wide markers; doesn’t require a reference genome; workflow can be adjusted to allow for barcoding (e.g. see Lemmon & Lemmon, 2012) | Markers are anonymous without a reference genome. | (Davey *et al.*, 2011, Lemmon & Lemmon, 2012) |
| Restriction-site-associated (RAD) DNA sequencing (RAD-seq) | DNA is fragmented using a restriction enzyme (usually rare cutter) and barcoded sequencing adaptors (P1) are attached to the ends of fragments. Fragments from all samples are pooled and the amount of genome to be sequenced is reduced by further random fragmentation and size selection. P2 adapters are ligated to all fragments. Only fragments containing restriction-sites (via presence of both P1 and P2 adaptors) are amplified and sequenced. | Obtain random, genome-wide markers; doesn’t require a reference genome; repeatable as a screening technique; amount of genome to be sequenced is reduced compared to RRLs giving greater coverage per region with equal sampling effort. If paired-end RAD-seq is used, haplotypes of each gene can be recovered. | Markers are anonymous without a reference genome. | (Baird *et al.*, 2008, Hohenlohe *et al.*, 2010, Davey *et al.*, 2011, Hohenlohe *et al.*, 2013) |
| Double-digest RAD-seq (ddRAD) | Modified RAD-seq approach. Uses two restriction enzymes to reduce amount of genome to be sequenced; eliminates the random fragmentation step; only fragments containing both restriction cut sites are amplified and sequenced | As per RAD-seq; but cheaper and more efficiently focuses given sequencing effort on the same regions among individuals, can start with less amount of DNA because minimising high DNA loss steps | As for RAD-seq; Technical issues relating to detection of PCR duplicates that cannot be resolved by using paired-end sequencing because of lack of random fragmentation step. | (Peterson *et al.*, 2012) |
| *Low coverage sequencing* | | | | |
| Genotyping-by-sequencing (GBS) | DNA is fragmented using a frequent-cutter restriction enzyme. Barcoded adaptors and common adaptors without barcodes are attached to ends of fragments. Only fragments containing a barcoded and a common adapter are amplified and sequenced. Because coverage is low, not all individuals will be genotyped at all sites. | Obtain random, genome-wide markers; useful for highly diverse taxa; simple (no size selection); compared with RAD is cheaper, fewer purification/selection steps. | Requires a reference genome or linkage map if imputing missing genotypes. | (Davey *et al.*, 2011, Elshire *et al.*, 2011) |
| MSG (multiplexed shotgun sequencing) | As per GBS, but uses only barcoded adapters (no common adapters) and fragments are size selected before sequencing. Because coverage is low, not all individuals will be genotyped at all sites. | As per GBS. | As per GBS. | (Andolfatto *et al.*, 2011, Davey *et al.*, 2011) |

**References**

Altshuler D, Durbin RM, Abecasis GR *et al.* (2010) A map of human genome variation from population-scale sequencing. *Nature,* 467, 1061-1073.

Andolfatto P, Davison D, Erezyilmaz D, Hu TT, Mast J, Sunayama-Morita T, Stern DL (2011) Multiplexed shotgun genotyping for rapid and efficient genetic mapping. *Genome Research,* 21, 610-617.

Baird NA, Etter PD, Atwood TS *et al.* (2008) Rapid SNP Discovery and Genetic Mapping Using Sequenced RAD Markers. *Plos One,* 3.

Bi K, Vanderpool D, Singhal S, Linderoth T, Moritz C, Good JM (2012) Transcriptome-based exon capture enables highly cost-effective comparative genomic data collection at moderate evolutionary scales. *Bmc Genomics,* 13.

Birol I, Raymond A, Jackman SD *et al.* (2013) Assembling the 20 Gb white spruce (Picea glauca) genome from whole-genome shotgun sequencing data. *Bioinformatics,* 29, 1492-1497.

Buerkle CA, Gompert Z, Parchman TL (2011) The n=1 constraint in population genomics. *Molecular Ecology,* 20, 1575-1581.

Cosart T, Beja-Pereira A, Chen SY, Ng SB, Shendure J, Luikart G (2011) Exome-wide DNA capture and next generation sequencing in domestic and wild species. *Bmc Genomics,* 12.

Davey JW, Hohenlohe PA, Etter PD, Boone JQ, Catchen JM, Blaxter ML (2011) Genome-wide genetic marker discovery and genotyping using next-generation sequencing. *Nature Reviews Genetics,* 12, 499-510.

Ellegren H, Smeds L, Burri R *et al.* (2012) The genomic landscape of species divergence in Ficedula flycatchers. *Nature,* 491, 756-760.

Elshire RJ, Glaubitz JC, Sun Q, Poland JA, Kawamoto K, Buckler ES, Mitchell SE (2011) A Robust, Simple Genotyping-by-Sequencing (GBS) Approach for High Diversity Species. *Plos One,* 6.

Everett MV, Grau ED, Seeb JE (2011) Short reads and nonmodel species: exploring the complexities of next-generation sequence assembly and SNP discovery in the absence of a reference genome. *Molecular Ecology Resources,* 11, 93-108.

Faircloth BC, Mccormack JE, Crawford NG, Harvey MG, Brumfield RT, Glenn TC (2012) Ultraconserved Elements Anchor Thousands of Genetic Markers Spanning Multiple Evolutionary Timescales. *Systematic Biology,* 61, 717-726.

Garvin MR, Saitoh K, Gharrett AJ (2010) Application of single nucleotide polymorphisms to non-model species: a technical review. *Molecular Ecology Resources,* 10, 915-934.

Gilad Y, Pritchard JK, Thornton K (2009) Characterizing natural variation using next-generation sequencing technologies. *Trends in Genetics,* 25, 463-471.

Grover CE, Salmon A, Wendel JF (2012) Targeted Sequence Capture as a Powerful Tool for Evolutionary Analysis. *American Journal of Botany,* 99, 312-319.

Helyar SJ, Hemmer-Hansen J, Bekkevold D *et al.* (2011) Application of SNPs for population genetics of nonmodel organisms: new opportunities and challenges. *Molecular Ecology Resources,* 11, 123-136.

Helyar SJ, Limborg MT, Bekkevold D *et al.* (2012) SNP Discovery Using Next Generation Transcriptomic Sequencing in Atlantic Herring (Clupea harengus). *Plos One,* 7.

Hohenlohe PA, Bassham S, Etter PD, Stiffler N, Johnson EA, Cresko WA (2010) Population Genomics of Parallel Adaptation in Threespine Stickleback using Sequenced RAD Tags. *Plos Genetics,* 6.

Hohenlohe PA, Day MD, Amish SJ *et al.* (2013) Genomic patterns of introgression in rainbow and westslope cutthroat trout illuminated by overlapping paired-end RAD sequencing. *Molecular Ecology,* 22, 3002-3013.

Jones FC, Grabherr MG, Chan YF *et al.* (2012) The genomic basis of adaptive evolution in threespine sticklebacks. *Nature,* 484, 55-61.

Lemmon AR, Emme SA, Lemmon EM (2012) Anchored Hybrid Enrichment for Massively High-Throughput Phylogenomics. *Systematic Biology,* 61, 727-744.

Lemmon AR, Lemmon EM (2012) High-Throughput Identification of Informative Nuclear Loci for Shallow-Scale Phylogenetics and Phylogeography. *Systematic Biology,* 61, 745-761.

Limborg MT, Helyar SJ, De Bruyn M *et al.* (2012) Environmental selection on transcriptome-derived SNPs in a high gene flow marine fish, the Atlantic herring (Clupea harengus). *Molecular Ecology,* 21, 3686-3703.

Mamanova L, Coffey AJ, Scott CE *et al.* (2010) Target-enrichment strategies for next-generation sequencing. *Nature Methods,* 7, 111-118.

Marko PB, Hart MW (2011) The complex analytical landscape of gene flow inference. *Trends in Ecology & Evolution,* 26, 448-456.

Metzker ML (2010) Applications of Next-Generation Sequencing Sequencing Technologies - the Next Generation. *Nature Reviews Genetics,* 11, 31-46.

Narum SR, Buerkle CA, Davey JW, Miller MR, Hohenlohe PA (2013) Genotyping-by-sequencing in ecological and conservation genomics. *Molecular Ecology,* 22, 2841-2847.

Peterson BK, Weber JN, Kay EH, Fisher HS, Hoekstra HE (2012) Double Digest RADseq: An Inexpensive Method for De Novo SNP Discovery and Genotyping in Model and Non-Model Species. *Plos One,* 7.

Zhang J, Chiodini R, Badr A, Zhang GF (2011) The impact of next-generation sequencing on genomics. *Journal of Genetics and Genomics,* 38, 95-109.
